# Biomass and Leaf Nutrition Contents of Selected Grass and Legume Species in High Altitude Rangelands of Kashmir Himalaya Valley (Jammu & Kashmir), India

**DOI:** 10.3390/plants12071448

**Published:** 2023-03-25

**Authors:** Javed A. Mugloo, Mehraj ud din Khanday, Mehraj ud din Dar, Ishrat Saleem, Hesham F. Alharby, Atif A. Bamagoos, Sameera A. Alghamdi, Awatif M. Abdulmajeed, Pankaj Kumar, Sami Abou Fayssal

**Affiliations:** 1 Division of Silviculture and Agro Forestry, Faculty of Forestry, Sher-e-Kashmir University of Agricultural Sciences and Technology of Kashmir, Kashmir 190025, India; drjaveed72@gmail.com (J.A.M.); mihraj.dar@gmail.com (M.u.d.D.); ishratsaleem1992@gmail.com (I.S.); 2 Division of Soil Science, Faculty of Horticulture, Sher-e-Kashmir University of Agricultural Sciences and Technology of Kashmir, Kashmir 190025, India; mehraj197@gmail.com; 3 Department of Biological Sciences, Faculty of Science, King Abdulaziz University, Jeddah 21589, Saudi Arabia; halharby@kau.edu.sa (H.F.A.); abamagoos@kau.edu.sa (A.A.B.); saalghamdy1@kau.edu.sa (S.A.A.); 4 Plant Biology Research Group, Department of Biological Sciences, Faculty of Science, King Abdulaziz University, Jeddah 21589, Saudi Arabia; 5 Biology Department, Faculty of Science, University of Tabuk, Umluj 46429, Saudi Arabia; awabdulmajeed@ut.edu.sa; 6 Agro-Ecology and Pollution Research Laboratory, Department of Zoology and Environmental Science, Gurukula Kangri (Deemed to Be University), Haridwar 249404, India; rs.pankajkumar@gkv.ac.in; 7 Department of Agronomy, Faculty of Agronomy, University of Forestry, 10 Kliment Ohridski Blvd, 1797 Sofia, Bulgaria; 8 Department of Plant Production, Faculty of Agriculture, Lebanese University, Beirut 1302, Lebanon

**Keywords:** biomass production, chemical composition, ecological management, ecosystem diversity, grazing

## Abstract

The yield and nutritional profile of grass and legume species in Kashmir Valley’s rangelands are scantly reported. The study area in this paper included three types of sites (grazed, protected, and seed-sown) divided into three circles: northern, central, and southern Kashmir. From each circle, three districts and three villages per district were selected. Most sites showed higher aboveground biomass (AGB) compared to belowground biomass (BGB), which showed low to moderate effects on biomass. The comparison between northern, central, and southern Kashmir regions revealed that AGB (86.74, 78.62, and 75.22 t. ha^−1^), BGB (52.04, 51.16, and 50.99 t. ha^−1^), and total biomass yield (138.78, 129.78, and 126.21 t. ha^−1^) were the highest in central Kashmir region, followed by southern and northern Kashmir regions, respectively. More precisely, AGB and total biomass yield recorded the highest values in the protected sites of the central Kashmir region, whereas BGB scored the highest value in the protected sites of southern Kashmir region. The maximum yield (12.5 t. ha^−1^) recorded among prominent grasses was attributed to orchard grass, while the highest crude fiber and crude protein contents (34.2% and 10.4%, respectively), were observed for Agrostis grass. The maximum yield and crude fiber content (25.4 t. ha^−1^ and 22.7%, respectively), among prominent legumes were recorded for red clover. The highest crude protein content (33.2%) was attributed to white clover. Those findings concluded the successful management of Kashmir rangelands in protected sites, resulting in high biomass yields along with the considerable nutritional value of grasses and legumes.

## 1. Introduction

The Jammu and Kashmir (J and K) region (India) is well known for its alpine and subalpine pastures [1], locally named “Margs” or “Bahaks”. These pastures constitute a crucial ecological resource and play a significant role in the socioeconomic state of the Himalayas Valley. The total area of Kashmir pasturelands is around 9595 km^2^ [2]. These pasturelands are rural areas that involve around 97% of the population in the agricultural sector. The rearing of sheep, goats, and cattle constitutes the locals’ subsidiary occupation. In addition, a huge population of nomadic Bakerwals, Gujjars, Chopans, Changpas, and Gaddies depends directly on meadow products and pasturelands for herds of maintained livestock.

The term “grassland” (also designated by “pastureland”) can be defined as land (and the vegetation growing on it) devoted to the production of introduced or indigenous forage for harvest via grazing, cutting, or both. The grassland’s vegetation includes grasses, legumes, and scantly woody species [3]. Thus, grassland is a highly dynamic ecosystem that supports fauna, flora, and human populations worldwide. It also encloses fodder crops that covered approximately 3.5 billion hectares in 2000 and contains around 20% of the world’s soil carbon stocks [4]. These stocks can be well enriched through the good management of grasslands via Voisin’s rational grazing (VRG), resulting in an increased milk production by ruminants [5]. This system allows the maximization of pasture growth associated with ruminant intake while maintaining a sustainable circular cycle [6]. The adoption of such a system is a must, especially in the Himalayan pasturelands, which was a scene of overgrazing over decades and centuries [7]. The literature reported a decrease in the available grazing area in the alpine and subalpine pasturelands of Kashmir from 0.15 ha. animal^−1^ to 0.10 ha. animal^−1^ between 1977 and 1982 [8]. Unfortunately, it is still at a continuous decrease rate according to a recent report [9].

As the globe is still facing a rise in climatic change, there is an increased demand to monitor, forecast and predict its effect on the productivity and quality of pasturelands [10]. However, the prediction of pasturelands’ biomass is sometimes not very reliable, while the accurate and precise estimation consists of traditional methods that are mainly costly, time-consuming, and non-environmentally friendly. In addition to this, the traditional influencing factors (i.e., high wind velocity, low temperature, snowstorms, and so on) and the increase in solar and ultraviolet (UV) radiations—as a direct response to climate change—have resulted in the decreased biomass production of Himalaya pasturelands [11]. Similar observations were acknowledged in Nepal [12] and in the African pasturelands of Niger and Zambia [10,13]. Thus, since the crucial role of pastureland in ecosystem functioning and soil stability is endangered, the safeguarding of the Himalayan alpine and subalpine vegetation exerts its weight [14].

Although earlier studies focused on the aboveground herbaceous species production in a very limited area in Kashmir Valley [15], none have explored the whole region’s above and belowground biomass (AGB and BGB, respectively), nor documented reliably the prominent grasses and legumes species growing there. These grasses and legumes are the main diet for sheep, goats, and cattle reared in the region. Thus, as the local population mainly relies on milk production, the compositional quality evaluation of biomass used for sheep, goats, and cattle nutrition is crucial. Therefore, the current study aimed to (a) investigate and estimate the biomass yield and leaf nutritional profile of grasses and legumes in high-altitude pasturelands of the northern, central and southern Kashmir Himalaya Valley (Jammu and Kashmir (J&K)), India; and (b) detect any possible effect (danger i.e., overgrazing) on grassland biomass in the studied zones.

## 2. Results

### 2.1. Analysis of Above (AGB), Below Ground Biomass (BGB) and Total Biomass Yield in Northern Kashmir

In northern Kashmir region, the average AGB/BGB ratio was the highest in the grazed and seed-sown sites of Kupwara district (1.61 and 1.85, respectively), and in the protected sites of Baramulla district (1.38) (Table 1). The average ratio of protected sites biomass over grazed sites biomass (R1) was comparable between districts, whereas the average ratio of seed-sown sites’ biomass over grazed sites’ biomass (R2) was the highest in Kupwara district. The percentages of villages within districts, where AGB > BGB (P1), and seed-sown sites biomass > grazed sites biomass (P3), were the highest in Kupwara district (100%). All districts showed a comparable percentage (100%) of villages where protected sites biomass > grazed sites biomass (P2).

All northern Kashmir districts showed higher AGB by 32.0%–43.2% in grazed sites compared to BGB, except in Firozpur (Baramulla district) and Ketsan (Bandipora district) where BGB was higher (*p* < 0.05) by 40.0% than AGB (Table 2). In protected sites, AGB was also higher (*p* < 0.05) by a range of 1.4%–40.3% than BGB, except in Ketsan (Bandipora district) where the latter was higher (*p* < 0.05) by 12.3% than the former. In all northern Kashmir districts, seed-grown sites showed higher (*p* < 0.05) AGB by a range of 20.1%–56.4% than BGB, whereas BGB was more abundant than AGB (*p* < 0.05) by 15.3% and 12.1% in Firozpur (Baramulla district) and Ketsan (Bandipora district), respectively. It was also depicted that the average biomass was higher (*p* < 0.05) for AGB than BGB in all districts (24.4%–43.2%), except for Firozpur (Baramulla district) and Ketsan (Bandipora district) (14.8% and 18.6%, respectively). Rajwar (Kupwara district), Gulmarg (Baramulla district), and Cithernaar (Bandipora district) showed the highest AGB and BGB among all districts and studied sites of northern Kashmir region. On the other hand, Gulmarg (Baramulla district) had the highest (*p* < 0.05) AGB among all studied sites, while Rajwar (Kupwara district) and Cithernaar (Bandipora district) had the highest (*p* < 0.05) BGB.

### 2.2. Analysis of Above (AGB), Below Ground Biomass (BGB) and Total Biomass Yield in Central Kashmir

In central Kashmir region, the average AGB/BGB ratio was the highest in all studied sites of Budgam district (2.08, 1.72, and 2.11 at grazed, protected and seed-sown sites, respectively), (Table 3). R1 was comparable between Ganderbal and Srinagar districts (2.70), whereas R2 was the highest in Srinagar district (1.53). P1, P2, and P3 were comparable between all central Kashmir districts (100%).

All central Kashmir districts showed a higher AGB (*p* < 0.05) by 19.7%–70.2% in grazed sites than BGB (Table 4). In protected sites, AGB was also higher (*p* < 0.05) by a range of 12.8%–47.9% than BGB. In all central Kashmir districts, seed-grown sites showed higher (*p* < 0.05) AGB by a range of 18.7%–57.1% than BGB. Moreover, the average biomass was higher (*p* < 0.05) for AGB than BGB in all districts (20.8%–55.6%). Narang (Ganderbal district), Kanidajan (Budgam district), and Chirenbal (Srinagar district) showed the highest AGB and BGB among all districts and studied sites of central Kashmir region. On the other hand, Kanidajan (Budgam district) showed significantly higher (*p* < 0.05) AGB and BGB in all sites except protected ones.

### 2.3. Analysis of Above (AGB), Below Ground Biomass (BGB) and Total Biomass Yield in Southern Kashmir

In southern Kashmir region, the average AGB/BGB ratio was the highest in the grazed and seed-sown sites of Anantnag district (1.71 and 1.82, respectively), and the protected sites of Shopian district (1.38) (Table 5). R1 and R2, and P1 and P3 scored the highest values in Anantnag and Kulgam districts, respectively, (2.38 and 1.47, and 100%, respectively). P3 was comparable between all southern Kashmir districts (100%).

Particularly, all southern Kashmir districts showed a higher AGB (*p* < 005) by 31.6%–51.9% in grazed sites than BGB, except in Dabjan (Shopian district) and Astanmarg (Kulgam district) where BGB was higher (*p* < 0.05) by 30.6% and 42.0% than AGB, respectively (Table 6). The same trend was observed regarding protected sites where AGB were higher (*p* < 0.05) by a range of 2.0%–40.3% than BGB, except in Dabjan (Shopian district) and Astanmarg (Kulgam district) where BGB was higher (*p* < 0.05) by 5.4% and 18.0% than AGB, respectively. In all southern Kashmir districts, seed-grown sites showed higher (*p* < 0.05) AGB by a range of 17.4%–56.6% than BGB, whereas BGB was more abundant (*p* < 0.05) by 15.3% and 12.8% than AGB in Dabjan (Shopian district) and Astanmarg (Kulgam district), respectively. Also, the average biomass was higher (*p* < 0.05) for AGB over BGB in all districts (14.9%–44.3%), except for Dabjan (Shopian district) and Astanmarg (Kulgam district) (15.1% and 22.0%, respectively). Aru Valley (Anantnag district), Kaller (Shopian district), and Chirenbal (Kulgam district) showed the highest AGB and BGB among all districts and studied sites. On the other hand, AGB was the highest (*p* < 0.05) in Kaller (Shopian district) in grazed and protected sites, and in Chirenbal (Kulgam district) among seed-sown sites. BGB was the highest (*p* < 0.05) in Chirenbal (Kulgam district) in grazed and seed-grown sites, and in Aru Valley (Anantnag district) in protected sites.

### 2.4. Comparison of Above (AGB), Belowground Biomass (BGB) and Total Biomass Yield between Kashmir Regions

**Figure 1:**
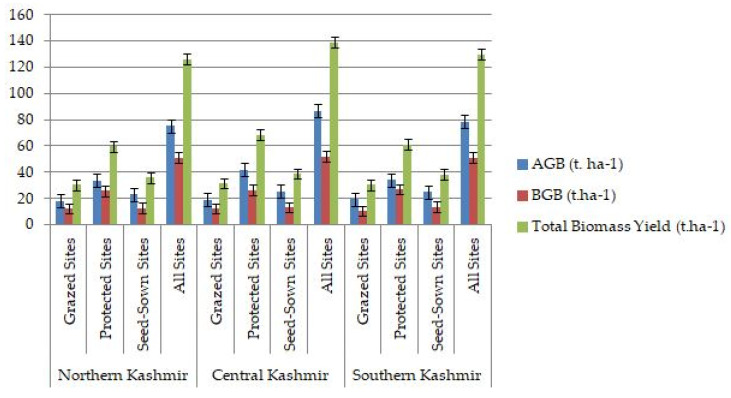
AGB, BGB and total biomass yield (t. ha^−1^) in studied circles.

The comparison between northern, central, and southern Kashmir regions revealed that AGB (86.74, 78.62, and 75.22 t. ha^−1^), BGB (52.04, 51.16, and 50.99 t. ha^−1^), and total biomass yield (138.78, 129.78, and 126.21 t. ha^−1^) were the highest in central Kashmir region, followed by southern and northern Kashmir ones, respectively, (Figure 1). More precisely, AGB and total biomass yield recorded the highest values in the protected sites of central Kashmir region (42.04 and 68.41 t. ha^−1^, respectively), whereas BGB scored the highest value in the protected sites of southern Kashmir region (27.00 t. ha^−1^).

### 2.5. Yield and Nutrient Profile of Prominent Grasses and Legumes

The results of prominent grasses’ yield and nutrient profile evaluation are shown in Table 7. Orchard grass (*Dactylis glomerata*) showed the highest yield (*p* < 0.05) (12.50 ± 0.6 t. ha^−1^) compared to other grasses, whereas, the highest nitrogen content (*p* < 0.05) was detected in Timothy grass (1.81 ± 0.05%). Phosphorus and crude protein contents were the most abundant (*p* < 0.05) in Agrostis grass (*Agrostis alba*) (0.34 ± 0.04% and 10.40 ± 0.5%, respectively), whereas perennial ryegrass (*Lolium perenne*) enclosed the highest (*p* < 0.05) potassium and crude fiber contents (0.44 ± 0.05% and 35.12 ± 0.8%, respectively). It should be noted that no significant difference (*p* > 0.05) and very low standard deviations (SDs) (0.2 < SD < 0.6, and 0.01 < SD < 0.8) were observed in terms of grass yield, and leaf nutrient content, respectively, between all studied sites in the three Kashmir regions.

Table 8 showed that red clover (*Trifolium pretense*) had the highest (*p* < 0.05) yield, nitrogen, potassium, and crude protein contents (25.40 ± 0.6 t. ha^−1^, 1.69 ± 0.05%, 0.45 ± 0.05%, and 22.70 ± 0.6%, respectively), among prominent legumes found in Kashmir Valley, whereas the phosphorus content was the most abundantly found (*p* < 0.05) in white clover (*Trifolium repens*) and sainfoin (*Onobrychis viciifolia*) (0.34 ± 0.04%). In addition, the crude fiber content scored its highest value in white clover (33.12 ± 0.8%) compared to other prominent legumes studied. It should be noted that no significant difference (*p* > 0.05) and very low SDs (0.3 < SD < 0.6, and 0.02 < SD < 0.8) were observed in terms of legume yield and leaf nutrient content, respectively, between all studied sites in the three Kashmir regions.

## 3. Discussion

### 3.1. Analysis of Above (AGB), Belowground Biomass (BGB), and Total Biomass Yield

The healthy functioning of an ecosystem can be estimated by the overall plant biomass it yields. Over-grazing is one of the most critical issues facing plant biomass in Kashmiri grasslands. The current study detected higher AGB compared to BGB and a positive AGB/BGB ratio in all districts of northern, central, and southern Kashmir regions. This simulates that the studied locations were not over-grazed. However, the high BGB in Aru Valley, Kaller and Chirenbal (northern Kashmir), Rajwar, Gulmarg, and Cithernaar (southern Kashmir), and Naranag, Kanidajan, and Chirenbal (central Kashmir) might reveal possible moderate grazing within these regions. Such regions might be abandoned after being heavily grazed in elder decades. In this regard, Dai et al. [16] reported that the moderate grazing promoted the root biomass of *Kobresia* meadow (BGB) in the northern Qinghai-Tibet pastures, which corroborates with our findings. Thus, the adaptive response of plants might occur in which they tend to increase their root development to survive [17]. Also, during the April-May period, snow melting occurs in the pasturelands of Kashmir Valley which naturally favors and promotes the growth and development of BGB. Furthermore, the protected areas (sites) by local authorities may have helped in the preservation of plant biomass away from rearing and over-grazing, thus resulting in increased biomass yields. This is consistent with the report of Lone and Pandit [18] on the Langate Forest division of Kashmir. Tittonell et al. [19] proposed an agroecological research agenda, suggesting species breeding to preserve diversity and a co-innovation of large-scale farming with farmers, policymakers, and value chains. Although site protection resulted in substantial improvements in rangeland grazing management in Namibia, it did not enhance cattle productivity nor rangeland health [20]. The interesting observation in the present study is that all studied species were found in grazed, protected, and seed-sown sites, which outlines again that grasslands were not over-grazed in the studied locations. Two decades back, it was reported that dry matter biomass yield ranged between 1.41 and 6.23 t. ha^−1^ in temperate pastures of the northwestern Himalayas [21], which is far below that observed in the current study. This could be explained by the fact that Kashmiri citizens are now more conscious and aware of the risks on grassland biomass and the disequilibrium of the environmental balance associated with overgrazing. On the other hand, climatic contrasts, species heterogeneity, and anthropogenic disturbances were reported to affect the biomass yield in the lesser Himalayan foothills, and northwestern regions of Kashmir Valley [22,23]. This simulates a possible inclusion of non-native species to the studied sites, resulting in the variation of soil organic carbon (SOC), and thus a variation in biomass yields [24]. Our findings outlined that AGB, BGB, and total biomass yield were the highest in central Kashmir, followed by southern and northern Kashmir. Such a variation in biomass yields was reported to be correlated with the variation in carbon sequestration potential (CSP) [25]. Panwar et al. [25] reported a high CSP in northern India (J & K as a whole state), associated with high biomass yields (AGB: 6.7–159.4 t. ha^−1^; BGB: 1.6–71.5 t. ha^−1^; total biomass yield: 15.9–202.6 t. ha^−1^). However, the study was very general and did not take into consideration the difference between Kashmiri regions in terms of vegetative populations nor site types (grazed, protected and seed-sown sites). Despite that, the AGB, BGB, and total biomass yields observed in the present study fall within the ranges stated in the aforementioned ones.

### 3.2. Yield and Nutrient Profile of Prominent Grasses and Legumes

The nutritional composition of grasses used for animal forage is a factor determining their growth, reproduction, and livestock production. Additionally, the climate, soil type, and degree of maturity of grasses influence their nutritional composition [26,27]. Sampling was performed during July which means that the studied grasses and legumes were at their harvest stage [28]. This simulates that their nutrient richness may have started to decline [29]. On the other hand, Hao and He [30] outlined an increased biomass yield once a nutrient loss occurs. This statement partly agrees with our findings as biomass yield was satisfying while grasses were highly nutritious. Leaf N, P, and K contents in orchard grass was several folds higher than outlined in other grasslands (N: 0.22–0.26%, *p*: 0.002–0.004%, K: 0.007–0.02%) [31]. A previous study on Chinese seed-sown pastures outlined leaf P and K contents in tall fescue grass higher by 1.7-fold and 4.9-fold than our findings [32]. Generally, leaf nitrogen content in grasses should not exceed 3.5% [33]; thus, our findings showed safe values. Leaf phosphorus content in grasses does not usually exceed 0.7–0.8% [34], which agrees with our findings, whereas leaf potassium content can range between 1.2 and 2.0% [35]. This simulates that the selected grasses may show some K deficiency. Moreover, Chang et al. [32] depicted a crude protein content in the range of 11–14%, being 1.5–1.9-fold higher than observed in the present study. On the other hand, the crude protein and crude fiber contents in selected legumes was promising. It was recommended that 12–19% of crude protein would be suitable for cattle feed [36]; which means that some of the selected grasses can be mixed with red or white clover to improve the protein requirements for rearing cattle. Thus, the high management of grasslands is proposed to increase the crude protein and crude fiber contents in selected grasses. In this context, Berauer et al. [37] reported that crude protein in grasses increased by 22–30% after high land management. Furthermore, the moderate nitrogen percentages in the studied grasses revealed the absence of an over-grazing activity in the studied sites. Dong et al. [38] explained that increased N rates, N mineralization, and nitrification processes occur when associated with over-grazing. It is worth noting that animals have different behaviors based on their preference. For instance, cattle and sheep diets are mainly based on grasses and legumes while goats’ diet is more related to herbal biomass [39]. Therefore, the present grasslands studied enclose nutritious grasses and legumes highly abundant for cattle and sheep foraging. This cannot be achieved without the inhibition of biomass species’ eradication unless correct and serious management of lands is performed associated with new technology that is timesaving and has a lower negative impact on the environment.

## 4. Materials and Methods

### 4.1. Study Area and Sites Description

The study area enclosed the whole Jammu and Kashmir (J and K) Valley, which was divided into three zone circles: northern, central, and southern. Within each circle, three districts were selected, and subsequently, three sites within each district were chosen (Table 9). All studied sites varied in elevation between 1450 and 4800 m above mean sea level. Studied areas enclosed: (a) Grazed Sites, (b) Protected Sites, and (c) Seed-Sown Sites. Grazed sites are grassland areas covered by grasses and legumes that are suitable for livestock grazing. Protected sites are grassland areas where carbon emissions from land use change are limited, and nutrient sapping is avoided. This included the cultivation of trees as windbreaks to reduce soil erosion and crop rotation to keep good nutrient availability in soil. Seed-sown sites are grassland areas which were seeded with native engendered species due to overgrazing or extensive agricultural exploitation. Seeded species included: orchard, tall fescue, perennial rye, and Agrostis grasses, and legumes such as: red clover, white clover, lucerne, sainfoin, and crown vetch. These species were sown in a randomized complete block design (RCBD) between mid-August and mid-September at a rate of 36 g seeds/m^2^. A harrowing process was also practiced for initial soil preparation in order to optimize seedling establishment.

### 4.2. Climate

The union territory of J and K, India (33°17′–37°20′ N latitude, 73°25′–80°30′ E longitude) comprises two main physical regions: Outer Himalayas with sub-tropical and intermediate climate (Jammu), and Inner Himalayas with a temperate climate (Kashmir). The climate varies considerably with altitude; it is mild and salubrious in lower altitudes but very cold in higher-ups. Spring is cool and rather wet. Regarding the Outer Himalayas (Jammu), average minimum and maximum temperatures vary between −11 °C and 33 °C during winter and summer, respectively. Autumn is bright and pleasant, while winter is extremely cold and experiences heavy snowfalls. Frost is experienced from the middle of November onwards. The main form of precipitation is snow in winter and some stray rains, and showering in spring. The Jammu region receives an average annual precipitation of about 1103 mm in the form of rain and snow for about 70 days. Unlike the Outer Himalayas, there is no distinct rainy season in the Inner Himalayas. The minimum temperature of the Kashmir region falls within −7 °C in winter and the maximum goes up to 35 °C in summer. It is characterized by a mean minimum temperature below 8 °C for more than six consecutive months per year. Mean annual minimum and maximum temperatures range between 6.68 and 19.31 °C, respectively, and mean annual soil temperature ranges between 8 and 15 °C, thus the area belongs to the mesic temperature regime. The mean annual rainfall in the Inner Himalayas is 710 mm and the soil in the studied area does not remain generally dry for more than 90 cumulative days. Hence, it belongs to the udic moisture regime.

### 4.3. Vegetation Diversity

Several types of grasses and legumes growing in the studied Kashmir regions are outlined in Table 10.

### 4.4. Sampling

Samples were collected following a direct field plot harvest method [40]. Briefly, three transects, divided into two blocks (100 m distant from each other) were performed. Moreover, three quadrants of 1 m^2^ each were performed within each block. All grasses and legumes, within these quadrants, were collected from their roots by digging 5 cm^2^ pits up to a depth of 30 cm. Then, they were packed in ice-cooled-bags, transported directly to the laboratory for identification, and stored at a cool temperature for further analyses.

### 4.5. Compositional Analyses

Before analyses, the vegetative components including roots were washed thoroughly under a jet of running tap water to remove the attached soil. Then, they were dipped in diluted HCl (1 mL concentrated HCL.L^−1^ water) [41]. Further washing was performed with de-ionized water. Sampled species were first identified as grasses or legumes, then the above- and belowground biomass yields were estimated in t. ha^−1^ [41]. Afterward, the roots were removed, and the clean leaf samples were dried in a hot air-circulating oven at 105 ℃ for 24 h until a constant weight is obtained. Then, they were girded for nutrient analysis [41].

The nitrogen content was estimated using the micro Kjeldahl method [42]. The phosphorus content was determined following the vanado-molybdo-phosphoric Acid yellow colorimetric method [43]. Briefly, the dissolved reactive phosphorus reacted with ammonium molybdate under acid conditions. Hence, the molybdo-phosphoric acid was formed, and a yellow vanado-molybdo-phosphoric acid was obtained in the presence of vanadium. This corresponds to the phosphorus concentration. Such concentration was detected at a wavelength of 470 nm using Thermo Spectronic Helios Gamma UV (Hellma model: 178.712-QS, flow cell: 10 mm light path, inner optical volume: 30 L), connected to a Kipp & Zonen BD112 recorder [43].

The potassium content was determined using the FLAPHO flame photometer method [44]. Briefly, the instrument was warmed up for 10 min and distilled water was fed to the instrument. Then, the indicators were adjusted to 0 (reading). The concentrated standard solution was aspirated, and the readout was adjusted to 90 (on the uppermost scale). Afterward, the distilled water was aspirated, and the instrument read 0. All standards and standard solutions were aspirated, and results were recorded. Then, the calibration curves were drawn; the potassium concentration corresponded to the abscissa, whereas the instrument readouts corresponded to the ordinate. Finally, the potassium concentration was noted.

Using the acid–alkali digestion method, the residue after acid and alkaline digestion (determined gravimetrically) corresponded to the crude fiber content [45]. The crude protein content was calculated after the determination of the leaf nitrogen via the micro Kjeldahl method. The leaf nitrogen content value was multiplied by a coefficient factor of 6.25 [46]. All compositional elements were expressed as percentage (%) dry matter.

### 4.6. Statistical Analysis

One-way ANOVA, Duncan, and Student’s t tests were applied for data analysis using the SPSS 25^®^ program. A confidence level of 95% (*p* = 0.05) was adopted for all statistical tests. A comparison between regions districts was performed (different lower-case letters refer to a significant difference) in terms of AGB/BGB, R1, R2, P1, P2, and P3 (Table 1, Table 3, and Table 5). A comparison between villages of different districts was performed (different capital letters refer to a significant difference) as well as between AGB and BGB (different lowercase letters refer to a significant difference (Table 2, Table 4, and Table 6). In a similar vein, a comparison between grasses and legume types in terms of yield, and compositional elements (N, P, K, crude fiber and crude protein), was performed (different letters refer to a significant difference) (Table 7 and Table 8).

## 5. Conclusions

Kashmir Valley rangelands (northern, central and southern Kashmir regions) were investigated for their AGB, BGB, and total biomass (grasses/legumes) yields in three site types (grazed, protected, and seed-sown) along with their leaf nutritional profiles (N, P, K, crude fiber, and crude protein contents). Results showed an overall moderate grazing with a low to moderate effect on biomass. AGB, BGB, and total biomass yields were the highest in central Kashmir, followed by southern, and northern Kashmir. AGB and total biomass yields recorded the highest values in the protected sites of central Kashmir region, whereas, BGB yield scored the highest value in the protected sites of southern Kashmir region. On the other hand, Agrostis grass showed the highest crude fiber and crude protein contents among grasses found in the studied regions, whereas the highest crude fiber and crude protein contents among prominent legumes were recorded for red clover and white clover, respectively. Those findings concluded the successful management of Kashmir rangelands in protected sites, resulting in high biomass yields along with the considerable nutritional value of grasses and legumes.

## Figures and Tables

**Table 1:** Comparison between northern Kashmir districts in terms of biomass production.

Parameter	Kupwara			Baramulla			Bandipora		
	Grazed Sites	Protected Sites	Seed-Sown Sites	Grazed Sites	Protected Sites	Seed-Sown Sites	Grazed Sites	Protected Sites	Seed-Sown Sites
AGB/BGB	1.61a	1.32b	1.85a	1.31b	1.38a	1.65c	1.29b	1.15c	1.81b
R1	2.37a			2.33a			2.36a		
R2	1.48a			1.12b			1.43b		
P1 (%)	100.00a			77.78b			66.67c		
P2 (%)	100.00a			100.00a			100.00a		
P3 (%)	100.00a			33.33c			66.67b		

AGB: above-ground biomass; BGB: below-ground biomass; R1: average ratio of protected sites biomass over grazed sites biomass (protected sites biomass/grazed sites biomass); R2: average ratio of seed-sown sites biomass over grazed sites biomass (seed-sown sites biomass/grazed sites biomass); P1: percentage of villages within district where ABG > BGB; P2: percentage of villages within district where protected sites biomass > grazed sites biomass; P3: percentage of villages within district where seed-sown sites biomass > grazed sites biomass. Values are means; means within the same row followed by different letters are significantly different at *p* ˂ 0.05 according to Duncan’s multiple range test.

**Table 2:** Above (AGB) and below Ground Biomass (BGB) (t/ha) in the pasture of northern Kashmir.

District	Village	Biomass Type	Grazed Sites	Protected Sites	Seed-Sown Sites	Average Biomass
Kupwara	Bangas Valley	AGB	0.75Da	1.46Da	1.34Ea	1.19Ea
		BGB	0.51Eb	1.06Gb	1.07Eb	0.80Eb
	Wogubal	AGB	1.11Ca	3.58Ca	1.81Ca	2.16Da
		BGB	0.63Db	2.53Cb	0.81Fb	1.32Cb
	Rajwar	AGB	4.26Ba	5.98Ba	4.83Aa	5.02Ba
		BGB	2.66Bb	5.17Ab	2.32Ab	3.38Ab
Baramulla	Firozpur	AGB	0.51Eb	1.38Ea	1.05Fb	0.98Fb
		BGB	0.85Ca	1.36Fb	1.24Da	1.15Da
	Dragbah	AGB	1.11Ca	3.58Ca	1.65Da	2.11Da
		BGB	0.63Db	2.43Db	0.85Fb	1.30Cb
	Gulmarg	AGB	4.46Aa	6.98Aa	4.81Aa	5.41Aa
		BGB	2.82Ab	4.17Bb	2.22Bb	3.07Bb
Bandipora	Ketsan	AGB	0.51Eb	1.28Fb	1.09Fb	0.96Fb
		BGB	0.85Ca	1.46Ea	1.24Da	1.18Da
	Awathwooth	AGB	1.11Ca	3.58Ca	1.95Ba	2.21Ca
		BGB	0.63Db	2.53Cb	0.85Fb	1.33Cb
	Cithernaar	AGB	4.26Ba	5.98Ba	4.81Aa	5.01Ba
		BGB	2.82Ab	5.17Ab	2.12Cb	3.37Ab

Values are means (3 replicates of each biomass type); means within the same column (comparison between villages in terms of AGB/BGB) followed by different capital letters are significantly different at *p* < 0.05 according to Duncan test; means within the same column (comparison between AGB and BGB within each village) followed by different lowercase letters are significantly different at *p* ˂ 0.05 according to Student’s t test.

**Table 3:** Comparison between central Kashmir districts in terms of biomass production.

Parameter	Ganderbal			Budgam			Srinagar		
	Grazed Sites	Protected Sites	Seed-Sown Sites	Grazed Sites	Protected Sites	Seed-Sown Sites	Grazed Sites	Protected Sites	Seed-Sown Sites
AGB/BGB	1.70b	1.46c	1.93b	2.08a	1.72a	2.11a	1.45c	1.57b	1.83c
R1	2.70a			2.62b			2.70a		
R2	1.47b			1.32c			1.53a		
P1 (%)	100.00a			100.00a			100.00a		
P2 (%)	100.00a			100.00a			100.00a		
P3 (%)	100.00a			100.00a			100.00a		

AGB: above-ground biomass; BGB: below-ground biomass; R1: average ratio of protected sites biomass over grazed sites biomass (protected sites biomass/grazed sites biomass); R2: average ratio of seed-sown sites biomass over grazed sites biomass (seed-sown sites biomass/grazed sites biomass); P1: percentage of villages within district where ABG > BGB; P2: percentage of villages within district where protected sites biomass > grazed sites biomass; P3: percentage of villages within district where seed-sown sites biomass > grazed sites biomass. Values are means; means within the same row followed by different letters are significantly different at *p* ˂ 0.05 according to Duncan’s multiple range test.

**Table 4:** Above (AGB) and belowground biomass (BGB) (t/ha) in the pasture of central Kashmir.

District	Village	Biomass Type	Grazed Sites	Protected Sites	Seed-Sown Sites	Average Biomass
Ganderbal	Rayil	AGB	0.85Ea	1.56Ga	1.34Fa	1.25Ga
		BGB	0.54Gb	1.36Fb	1.09Db	0.99Gb
	Wangeth	AGB	1.11Ca	4.58Da	1.95Da	2.54Ea
		BGB	0.63Fb	2.93Db	0.85Fb	1.47Fb
	Naranag	AGB	4.26Ba	6.98Ba	4.81Ba	5.35Ca
		BGB	2.42Cb	4.17Cb	2.13Cb	2.91Db
Budgam	Yousmarg	AGB	0.94Da	1.88Ea	1.19Ga	1.33Fa
		BGB	0.28Hb	0.98Hb	0.51Gb	0.59Hb
	Dodhpathri	AGB	1.18Ca	4.68Ca	1.98Da	2.61Da
		BGB	0.72Eb	2.97Db	0.87Fb	1.52Eb
	Kanidajan	AGB	4.76Aa	7.99Aa	5.88Aa	6.21Aa
		BGB	3.82Ab	4.80Bb	3.42Ab	4.01Ab
Srinagar	Astanmarg	AGB	0.73Fa	1.78Fa	1.41Ea	1.31Fa
		BGB	0.55Gb	1.16Gb	1.09Db	0.94Gb
	Zahgemarg	AGB	1.11Ca	4.61Ca	2.09Ca	2.61Da
		BGB	0.83Db	2.83Eb	0.95Eb	1.54Eb
	Chirenbal	AGB	4.26Ba	7.98Aa	4.85Ba	5.71Ba
		BGB	2.52Bb	5.17Ab	2.42Bb	3.37Bb

Values are means (3 replicates of each biomass type); means within the same column (comparison between villages in terms of AGB/BGB) followed by different capital letters are significantly different at *p* < 0.05 according to Duncan test; means within the same column (comparison between AGB and BGB within each village) followed by different lowercase letters are significantly different at *p* ˂ 0.05 according to the Student’s t-test.

**Table 5:** Comparison between southern Kashmir districts in terms of biomass production.

Parameter	Anantnag			Shopian			Kulgam		
	Grazed Sites	Protected Sites	Seed-Sown Sites	Grazed Sites	Protected Sites	Seed-Sown Sites	Grazed Sites	Protected Sites	Seed-Sown Sites
AGB/BGB	1.71a	1.18b	1.82a	1.31c	1.38a	1.69c	1.43b	1.13b	1.71b
R1	2.38a			2.19c			2.23b		
R2	1.43b			1.31c			1.47a		
P1 (%)	100.00a			66.67b			66.67b		
P2 (%)	100.00a			100.00a			100.00a		
P3 (%)	66.67b			66.67b			100.00a		

AGB: above-ground biomass; BGB: below-ground biomass; R1: average ratio of protected sites biomass over grazed sites biomass (protected sites biomass/grazed sites biomass); R2: average ratio of seed-sown sites biomass over grazed sites biomass (seed-sown sites biomass/grazed sites biomass); P1: percentage of villages within district where ABG > BGB; P2: percentage of villages within district where protected sites biomass > grazed sites biomass; P3: percentage of villages within district where seed-sown sites biomass > grazed sites biomass. Values are means; means within the same row followed by different letters are significantly different at *p* < 0.05 according to Duncan’s multiple range test.

**Table 6:** Above (AGB) and belowground biomass (BGB) (t/ha) in the pasture of southern Kashmir.

District	Village	Biomass Type	Grazed Sites	Protected Sites	Seed-Sown Sites	Average Biomass
Anantnag	Daksum	AGB	0.79Ga	1.51Fa	1.32Fa	1.21Ea
		BGB	0.54Fb	1.48Gb	1.09Eb	1.03Eb
	Alhan	AGB	1.25Ea	3.74Ca	1.98Da	2.32Ca
		BGB	0.65Eb	2.58Db	0.91Fb	1.38Cb
	Aru Valley	AGB	4.69Aa	5.98Ba	4.81Ba	5.16Ba
		BGB	2.68Bb	5.57Ab	2.32Bb	3.52Ab
Shopian	Dabjan	AGB	0.59Hb	1.41Gb	1.05Gb	1.01Fb
		BGB	0.85Ca	1.49Ga	1.24Da	1.19Da
	Hirpora	AGB	1.21Fa	3.68Da	1.85Ea	2.24Da
		BGB	0.77Db	2.43Eb	0.92Fb	1.37Cb
	Kaller	AGB	4.70Aa	6.98Aa	4.91Ba	5.51Aa
		BGB	2.82Ab	4.17Cb	2.22Cb	3.07Bb
Kulgam	Astanmarg	AGB	0.51Hb	1.28Hb	1.09Gb	0.96Gb
		BGB	0.88Ca	1.56Fa	1.25Da	1.23Da
	Zahgemarg	AGB	1.31Da	3.58Ea	2.05Ca	2.31Ca
		BGB	0.63Eb	2.53Db	1.05Eb	1.40Cb
	Chirenbal	AGB	4.56Ba	5.98Ba	5.81Aa	5.45Aa
		BGB	2.82Ab	5.19Bb	2.52Ab	3.51Ab

Values are means (3 replicates of each biomass type); means within the same column (comparison between villages in terms of AGB/BGB) followed by different capital letters are significantly different at *p* < 0.05 according to Duncan test; means within the same column (comparison between AGB and BGB within each village) followed by different lowercase letters are significantly different at *p*< 0.05 according to the Student’s t test.

**Table 7:** Yield and leaf nutrient profile of prominent grasses.

Grasses	Yield (t. ha^−1^)	N (%)	*p* (%)	K (%)	Crude Fiber (%)	Crude Protein (%)
*Dactylis glomerata* (Orchard grass)	12.50 ± 0.6a	1.51 ± 0.04b	0.07 ± 0.01d	0.31 ± 0.04c	15.30 ± 0.6b	10.30 ± 0.5a
*Festuca arundinacea* (Tall fescue grass)	11.80 ± 0.6ab	1.22 ± 0.03c	0.18 ± 0.02c	0.41 ± 0.05ab	11.20 ± 0.6b	7.50 ± 0.3b
*Lolium perenne* (Perennial rye grass)	10.23 ± 0.5b	1.29 ± 0.03c	0.29 ± 0.03b	0.44 ± 0.05a	35.12 ± 0.8a	5.10 ± 0.2c
*Phleum pratense* (Timothy grass)	8.57 ± 0.4c	1.81 ± 0.05a	0.21 ± 0.03c	0.39 ± 0.04b	31.20 ± 0.8a	9.50 ± 0.4a
*Agrostis alba* (Agrostis grass)	3.51 ± 0.2d	1.23 ± 0.03c	0.34 ± 0.04a	0.23 ± 0.03d	34.20 ± 0.8a	10.40 ± 0.5a

Values are means (3 replicates of each grass); means within the same column followed by different letters are significantly different at *p*< 0.05 according to Duncan’s multiple range test.

**Table 8:** Yield and leaf nutrient profile of prominent legumes.

Legumes	Yield (t. ha^−1^)	N (%)	*p* (%)	K (%)	Crude Fiber (%)	Crude Protein (%)
*Trifolium pratense* (Red clover)	25.40 ± 0.6a	1.69 ± 0.05a	0.33 ± 0.04a	0.45 ± 0.05a	29.89 ± 0.7a	22.70 ± 0.6a
*Trifolium repens* (White clover)	24.20 ± 0.6ab	1.23 ± 0.03c	0.34 ± 0.04a	0.25 ± 0.03c	33.12 ± 0.8a	21.10 ± 0.6a
*Medicago sativa L*. (Lucerne)	23.40 ± 0.6b	1.67 ± 0.05a	0.31 ± 0.04a	0.43 ± 0.05a	11.50 ± 0.6b	18.80 ± 0.5b
*Onobrychis viciifolia* (Sainfoin)	7.50 ± 0.3d	1.19 ± 0.02c	0.34 ± 0.04a	0.23 ± 0.03c	10.20 ± 0.5b	15.40 ± 0.5c
*Securigera varia* (Crown vetch)	9.30 ± 0.4c	1.47 ± 0.04b	0.11 ± 0.02b	0.35 ± 0.04b	9.23 ± 0.4b	11.56 ± 0.5d

Values are means (3 replicates of each grass); means within the same column followed by different letters are significantly different at *p*< 0.05 according to Duncan’s multiple range test.

**Table 9:** Spatial distribution of studied sites.

Zone Name	District Name	Village Name	District Coordinates	District Altitude
Northern Kashmir	Kupwara	Bangus Valley, Wogubal, Rajwar	34°18′–34°47′ N, 73°45′–74°30′ E	2000–3500 m
	Baramulla	Firozpur, Dragbah, Gulmarg	34°11′–34°19′ N, 74°21′–74°36′ E	1630–2085 m
	Bandipora	Ketsan, Awathwooth, Cithernaar	34°25′–34°41′ N, 74°39′–74°65′ E	2700–4800 m
Central Kashmir	Srinagar	Baedhmargh Reshipora, Astanmarg Dara, Syedpora Bla	34°05′–34°08′ N, 74°50′–74°83′ E	1450–3942 m
	Budgam	Yousmarg, Dodhpathri, Kanidajan	33°93′–34°02′ N, 74°69′–74°79′ E	2000–2730 m
	Ganderbal	Rayil, Wangeth, Naranag	33°44′–33°73′ N, 75°09′–75°15′ E	1716–3397 m
Southern Kashmir	Anantnag	Daksum, Ahlan, Aru Valley	33°36′–34°25′ N, 75°02′–75°59′ E	1600–1723 m
	Shopian	Dabjan, Hirpora, Kaller	33°43′–33°72′ N, 74°50′–74°83′ E	1650–4720 m
	Kulgam	Astanmarg, Zahgemarg, Chirenbal	33°55′–33°78′ N, 74°90′–75°17′ E	1740–4800 m

**Table 10:** Diversity of grasses and legumes in the studied Kashmir regions.

Zone	Grasses	Legumes
Northern Kashmir	*Dactylis glomerata*	*Trifolium pratense*
	*Festuca arundinacea*	*Trifolium repens*
	*Lolium perenne*	*Onobrychis viciifolia*
	*Phleum pratense*	*Medicago sativa*
	*Bromus unioloides*	*Securigera varia*
	*Phalaris* spp.	
	*Poa pratensis*	
	*Lolium multiflorum*	
	*Agrostis alba*	
	*Avena sativa*	
Central Kashmir	*Dactylis glomerata*	*Trifolium alexandrinum*
	*Festuca arundinacea*	*Stylosanthus hamata*
	*Lolium perenne*	*Macroptilium atropupreum*
	*Dicanthium annulatum*	*Trifolium pratense*
	*Chloris gayana*	*Trifolium repens*
	*Chrysopogon fulvus*	*Onobrychis viciifolia*
	*Heteropogon contortus*	*Medicago sativa*
	*Agrostis alba*	*Securigera varia*
	*Setaria* spp.	
	*Avena sativa*	
	*Phleum pratense*	
Southern Kashmir	*Dactylis glomerata*	*Trifolium pratense*
	*Festuca arundinacea*	*Trifolium repens*
	*Lolium perenne*	*Onobrychis viciifolia*
	*Agrostis alba*	*Medicago sativa*
	*Phleum pratense*	*Securigera varia*
	*Avena sativa*	
	*Setaria* spp.	

## Data Availability

All data used in the present study are included in the manuscript.

## References

[1] Haq S.M., Hassan M., Jan H.A., Al-Ghamdi A.A., Ahmad K., Abbasi A.M. (2022). Traditions for Future Cross-National Food Security–Food and Foraging Practices among Different Native Communities in the Western Himalayas. Biology.

[2] Singh J.P., Dev I., Deb D., Chaurasia R.S., Radotra S., Roy M.M., Malaviya D.R., Yadav V.K., Singh T., Sah R.P., Vijay D., Radhakrishna A. (2015). Identification and characterization of pastureland and other grazing resources of Jammu & Kashmir using GIS and satellite remote sensing technique. Proceedings of the XXIII International Grassland Congress, Sustainable Use of Grassland Resources for Forage Production, Biodiversity and Environmental Protection.

[3] Louhaichi M., Gamoun M., Hassan S., Abdallah M.A.B. (2021). Characterizing Biomass Yield and Nutritional Value of Selected Indigenous Range Species from Arid Tunisia. Plants.

[4] FAO (2015). Food and Agriculture Organization of the United Nations—Statistic Division (FAOSTAT). http://faostat3.fao.org/home/E.

[5] Seó H.L.S., Machado Filho L.C.P., Brugnara D. (2017). Rationally Managed Pastures Stock More Carbon than No-Tillage Fields. Front. Environ. Sci..

[6] Pinheiro Machado Filho L.C., Seó H.L.S., Daros R.R., Enriquez-Hidalgo D., Wendling A.V., Pinheiro Machado L.C. (2021). Voisin Rational Grazing as a Sustainable Alternative for Livestock Production. Animals.

[7] Apollo M., Andreychouk V., Bhattarai S. (2018). Short-Term Impacts of Livestock Grazing on Vegetation and Track Formation in a High Mountain Environment: A Case Study from the Himalayan Miyar Valley (India). Sustainability.

[8] Misri B. (2003). Improvement of Sub-Alpine and Alpine Himalayan Pastures. Research Centre.

[9] Qureshi R.A., Ghufran M.A., Gilani S.A., Sultana K., Ashraf M. (2007). Ethnobotanical studies of selected medicinal plants of Sudhan Gali and Ganga Chotti hills, district Bagh, Azad Kashmir. Pak. J. Bot..

[10] Clementini C., Pomente A., Latini D., Kanamaru H., Vuolo M.R., Heureux A., Fujisawa M., Schiavon G., Del Frate F. (2020). Long-Term Grass Biomass Estimation of Pastures from Satellite Data. Remote Sens..

[11] Sulzberger B., Austin A.T., Cory R.M., Zepp R.G., Paul N.D. (2019). Solar UV radiation in a changing world: Roles of cryosphere-land-water-atmosphere interfaces in global biogeochemical cycles. Photochem. Photobiol. Sci..

[12] Paudel K.P., Andersen P. (2010). Assessing rangeland degradation using multi temporal satellite images and grazing pressure surface model in Upper Mustang, Trans Himalaya, Nepal. Remote Sens. Environ..

[13] Schucknecht A., Meroni M., Kayitakire F., Rembold F., Boureima A. (2015). Biomass estimation to support pasture management in Niger. Int. Arch. Photogramm. Remote Sens. Spat. Inf. Sci..

[14] Kuniyal J.C., Maiti P., Kumar S., Kumar A., Bisht N., Sekar K.C., Arya S.C., Rai S., Nand M. (2021). Dayara bugyal restoration model in the alpine and subalpine region of the Central Himalaya: A step toward minimizing the impacts. Sci. Rep..

[15] Saleem I., Mugloo J.A., Anjum Baba A., Buch K. (2019). Biomass estimation of herbaceous species of Benhama area, Kashmir. J. Pharmacogn. Phytochem..

[16] Dai L., Guo X., Ke X., Zhang F., Li Y., Peng C., Shu K., Li Q., Lin L., Cao G. (2019). Moderate grazing promotes the root biomass in *Kobresia* meadow on the northern Qinghai-Tibet Plateau. Ecol. Evol..

[17] Qu K., Cheng Y., Gao K., Ren W., Fry E.L., Yin J., Liu Y. (2022). Growth-Defense Trade-Offs Induced by Long-term Overgrazing Could Act as a Stress Memory. Front. Plant Sci..

[18] Lone H.A., Pandit A.K. (2007). Impact of grazing on community features and biomass of herbaceous species in Langate Forest Division of Kashmir. Indian For..

[19] Tittonell P., Piñeiro G., Garibaldi L.A., Dogliotti S., Olff H., Jobbagy E.G. (2020). Agroecology in Large Scale Farming—A Research Agenda. Front. Sustain. Food Syst..

[20] Coppock D.L., Crowley L., Durham S.L., Groves D., Jamison J.C., Karlan D., Norton B.E., Ramsey R.D. (2022). Community-based rangeland management in Namibia improves resource governance but not environmental and economic outcomes. Commun. Earth Environ..

[21] Sharma B.R., Koranne K.D., Singh P., Pathak P.S. (1988). Present status and management strategies for increasing biomass production in North-Western Himalayan rangelands. Rangelands—Resources and Management.

[22] Dad J.M. (2019). Organic carbon stocks in mountain grassland soils of northwestern Kashmir Himalaya: Spatial distribution and effects of altitude, plant diversity and land use. Carbon Manag..

[23] Khan R.W.A., Shaheen H., Awan S.N. (2021). Biomass and soil carbon stocks in relation to the structure and composition of Chir Pine dominated forests in the lesser Himalayan foothills of Kashmir. Carbon Manag..

[24] Hoffmann U., Hoffmann T., Jurasinski G., Glatzel S., Kuhn N.J. (2014). Assessing the spatial variability of soil organic carbon stocks in an alpine setting (Grindelwald, Swiss Alps). Geoderma.

[25] Panwar P., Mahalingappa D.G., Kaushal R., Bhardwaj D.R., Chakravarty S., Shukla G., Thakur N.S., Chavan S.B., Pal S., Nayak B.G. (2022). Biomass Production and Carbon Sequestration Potential of Different Agroforestry Systems in India: A Critical Review. Forests.

[26] Ravhuhali K.E., Mlambo V., Beyene T.S., Palamuleni L.G. (2021). Effect of soil type on spatial distribution and nutritive value of grass species growing in selected rangelands of South Africa. S. Afr. J. Plant Soil.

[27] Mokgakane T.J., Mlambo V., Ravhuhali K.E., Magoro N. (2021). Contribution of Soil Type to Quantify and Nutritiional Value of Grass Species on the South African Highveld. Resources.

[28] Boob M., Elsaesser M., Thumm U., Hartung J., Lewandowski I. (2019). Harvest Time Determines Quality and Usability of Biomass from Lowland Hay Meadows. Agriculture.

[29] Mwangi F.W., Charmley E., Adegboye O.A., Gardiner C.P., Malau-Aduli B.S., Kinobe R.T., Malau-Aduli A.E.O. (2022). Chemical Composition and In Situ Degradability of *Desmanthus* spp. Forage Harvested at Different Maturity Stages. Fermentation.

[30] Hao Y., He Z. (2019). Effects of grazing patterns on grassland biomass and soil environments in China: A meta-analysis. PLoS ONE.

[31] Pavlů L., Gaisler J., Hejcman M., Pavlů V.V. (2016). What is the effect of long-term mulching and traditional cutting regimes on soil and biomass chemical properties, species richness and herbage production in Dactylis glomerata grassland?. Agric. Ecosyst. Environ..

[32] Chang S., Xie K., Du W., Jia Q., Yan T., Yang H., Hou F. (2022). Effects of Mowing Times on Nutrient Composition and In Vitro Digestibility of Forage in Three Sown Pastures of China Loess Plateau. Animals.

[33] Gislum R., Griffith S.M. (2005). Tiller Production and Development in Perennial Ryegrass in Relation to Nitrogen Use. J. Plant Nutr..

[34] Zhao X., Lyu Y., Jin K., Lambers H., Shen J. (2021). Leaf Phosphorus Concentration Regulates the Development of Cluster Roots and Exudation of Carboxylates in *Macadamia integrifolia*. Front. Plant Sci..

[35] Jin Y., Lai S., Chen Z., Jian C., Zhou J., Niu F., Xu B. (2022). Leaf Photosynthetic and Functional Traits of Grassland Dominant Species in Response to Nutrient Addition on the Chinese Loess Plateau. Plants.

[36] Katongole C.B., Yan T. (2020). Effect of Varying Dietary Crude Protein Level on Feed Intake, Nutrient Digestibility, Milk Production, and Nitrogen Use Efficiency by Lactating Holstein-Friesian Cows. Animals.

[37] Berauer B.J., Wilfahrt P.A., Reu B., Schuchardt M.A., Garcia-Franco N., Zistl-Schlingmann M., Dannenmann M., Kiese R., Kühnel A., Jentsch A. (2020). Predicting forage quality of species-rich pasture grasslands using vis-NIRS to reveal effects of management intensity and climate change. Agric. Ecosyst. Environ..

[38] Dong J., Tian L., Zhang J., Liu Y., Li H., Dong Q. (2022). Grazing Intensity Has More Effect on the Potential Nitrification Activity Than the Potential Denitrification Activity in An Alpine Meadow. Agriculture.

[39] Ayele J., Tolemariam T., Beyene A., Tadese D.A., Tamiru M. (2022). Biomass composition and dry matter yields of feed resource available at Lalo kile district of Kellem Wollega Zone, Western Ethiopia. Heliyon.

[40] Ray D.K., Sloat L.L., Garcia A.S., Davis K.F., Ali T., Xie W. (2022). Crop harvests for direct food use insufficient to meet the UN’s food security goal. Nat. Food.

[41] Ruan L., Cheng H., Ludewig U., Li J., Chang S.X. (2022). Root Foraging Strategy Improves the Adaptability of Tea Plants (*Camellia sinensis* L.) to Soil Potassium Heterogeneity. Int. J. Mol. Sci..

[42] Hicks T.D., Kuns C.M., Raman C., Bates Z.T., Nagarajan S. (2022). Simplified Method for the Determination of Total Kjeldahl Nitrogen in Wastewater. Environments.

[43] Desai S., Amaresan N., Dharumadurai D. (2022). Qualitative and Quantitative Estimation of Phosphate Solubilizing Actinobacteria. Methods in Actinobacteriology.

[44] Junsomboon J., Jakmunee J. (2011). Determination of potassium, sodium, and total alkalies in portland cement, fly ash, admixtures, and water of concrete by a simple flow injection flame photometric system. J. Anal. Methods Chem..

[45] Dhingra D., Michael M., Rajput H., Patil R.T. (2012). Dietary fibre in foods: A review. J. Food Sci. Technol..

[46] Liu T., Ren T., White P.J., Cong R., Lu J. (2018). Storage nitrogen co-ordinates leaf expansion and photosynthetic capacity in winter oilseed rape. J. Exp. Bot..

